# Supplementary material for: Context-dependent activation and evolutionary buffering of a mating pheromone in fission yeast
Source: Commun Biol. 2026 Apr 21;9:534. doi: 10.1038/s42003-026-10058-6 (PMC13099959; doi:10.1038/s42003-026-10058-6)
Supplement: Supplementary file 1 — Supplementary Information [file 42003_2026_10058_MOESM1_ESM.pdf]

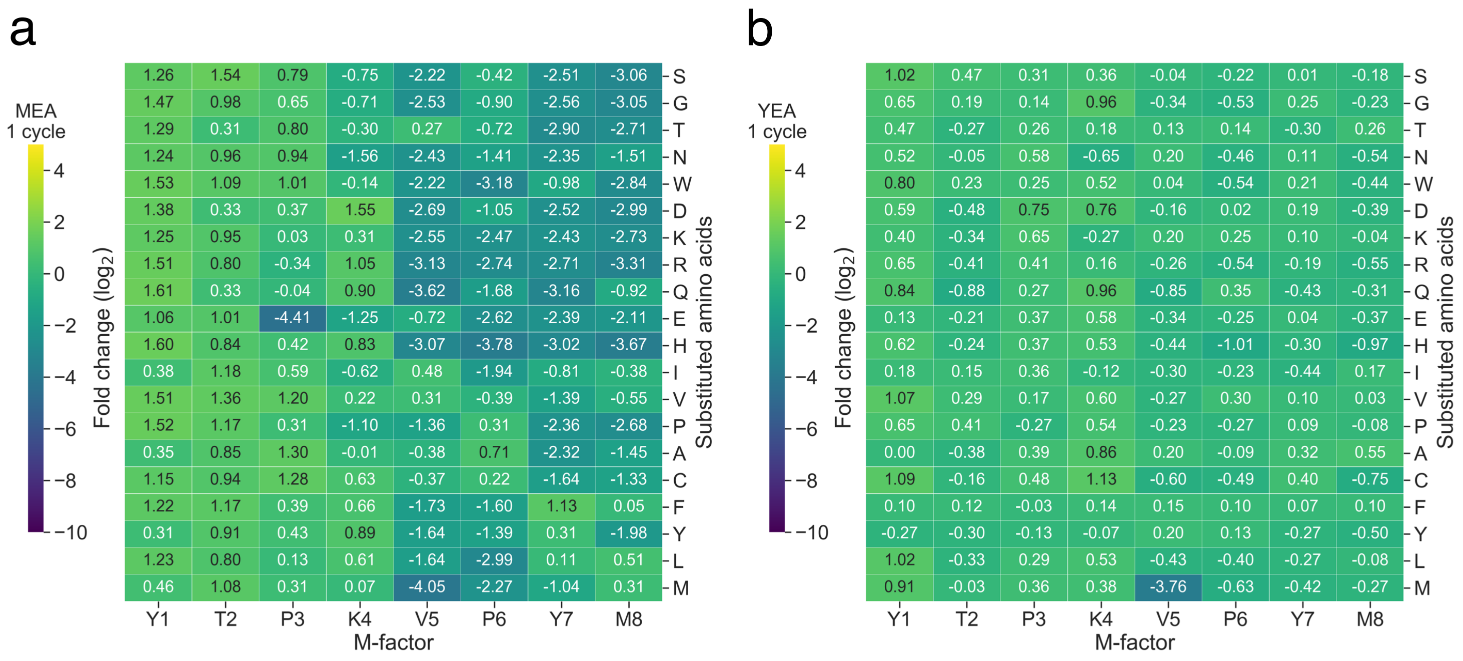

Supplementary Fig. 1 | One-cycle competition outcomes on MEA and YEA.

Pairwise scatter plots comparing $\log_2$ fold changes of all 153 strains between independent replicates. Each dot represents one strain; the diagonal line indicates y = x. High R^2 values demonstrate good reproducibility.

Supplementary Fig. 2

a

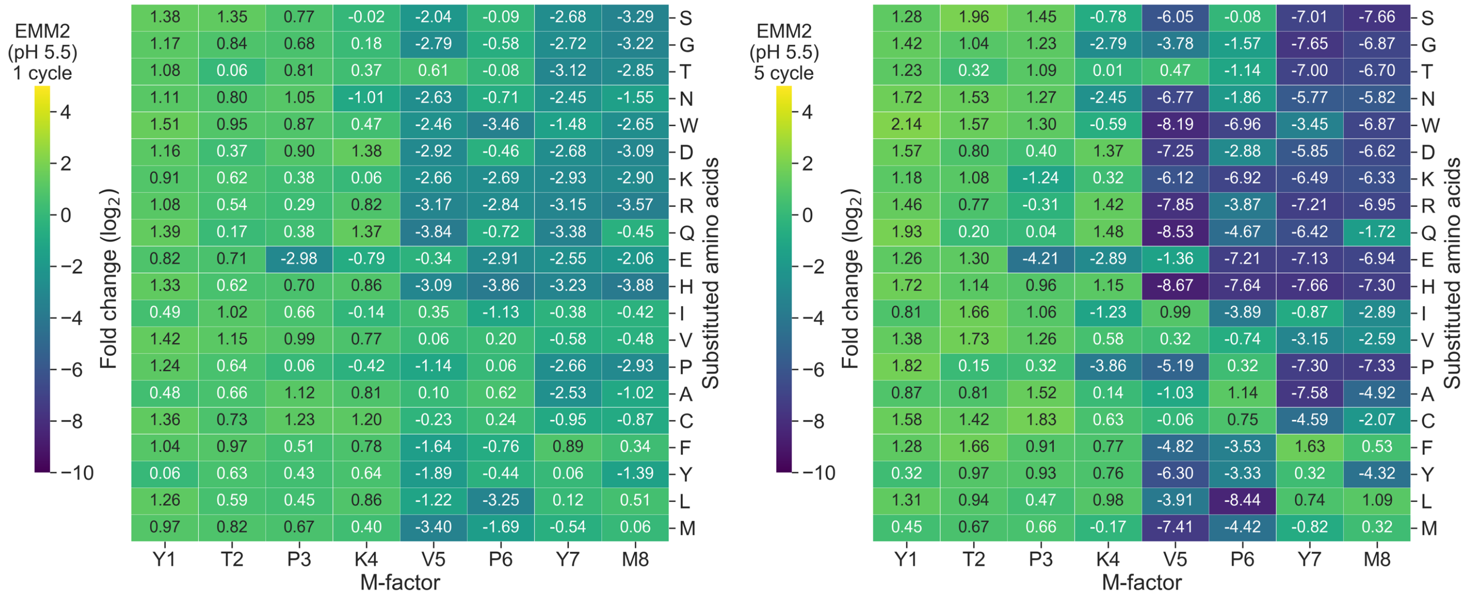

b

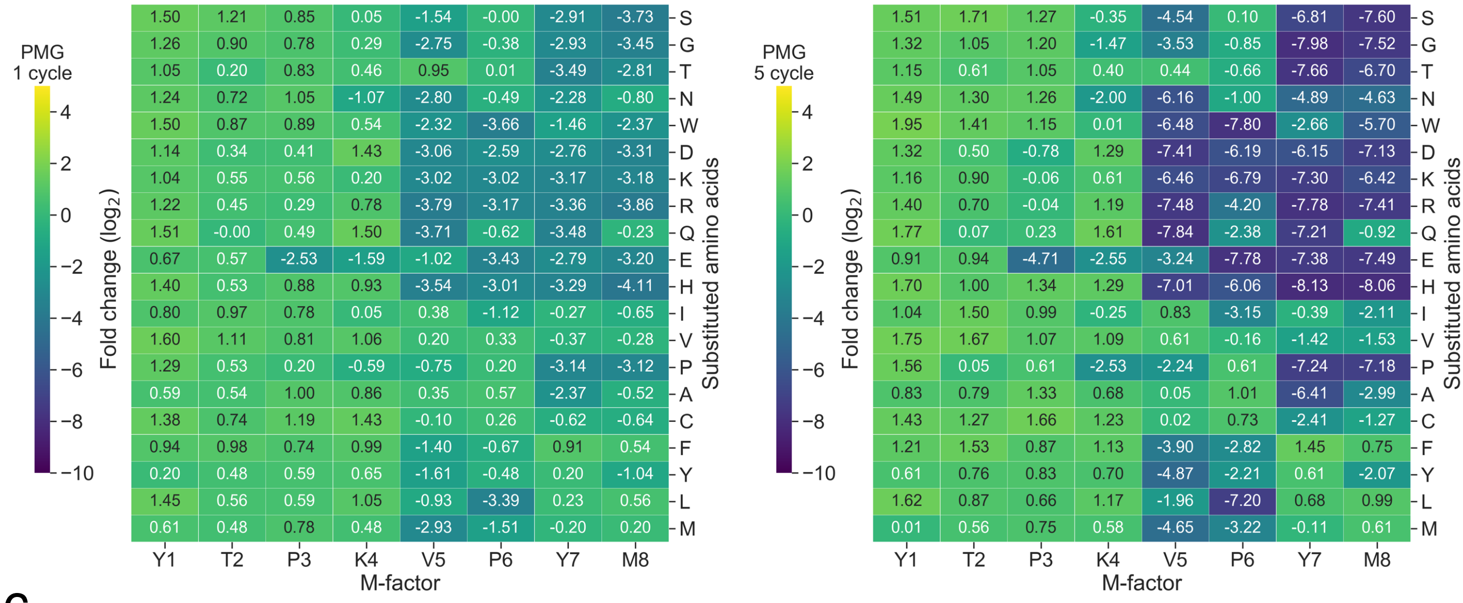

c

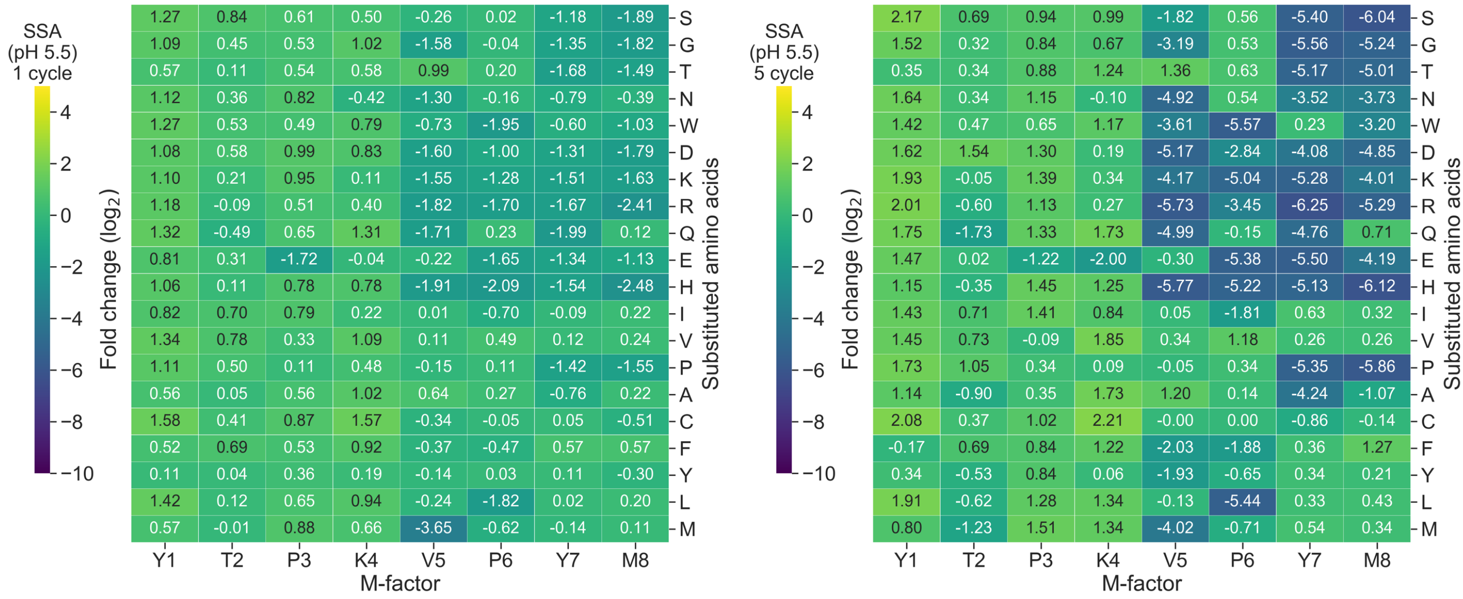

Supplementary Fig. 2 | Competition outcomes on EMM2 (pH 5.5), PMG, and SSA (pH 5.5).

Heatmaps showing log_2 fold change in strain abundance after mating–sporulation cycles 1 and 5 on (a) EMM2 (pH 5.5), (b) PMG, and (c) SSA (pH 5.5).

Supplementary Fig. 3

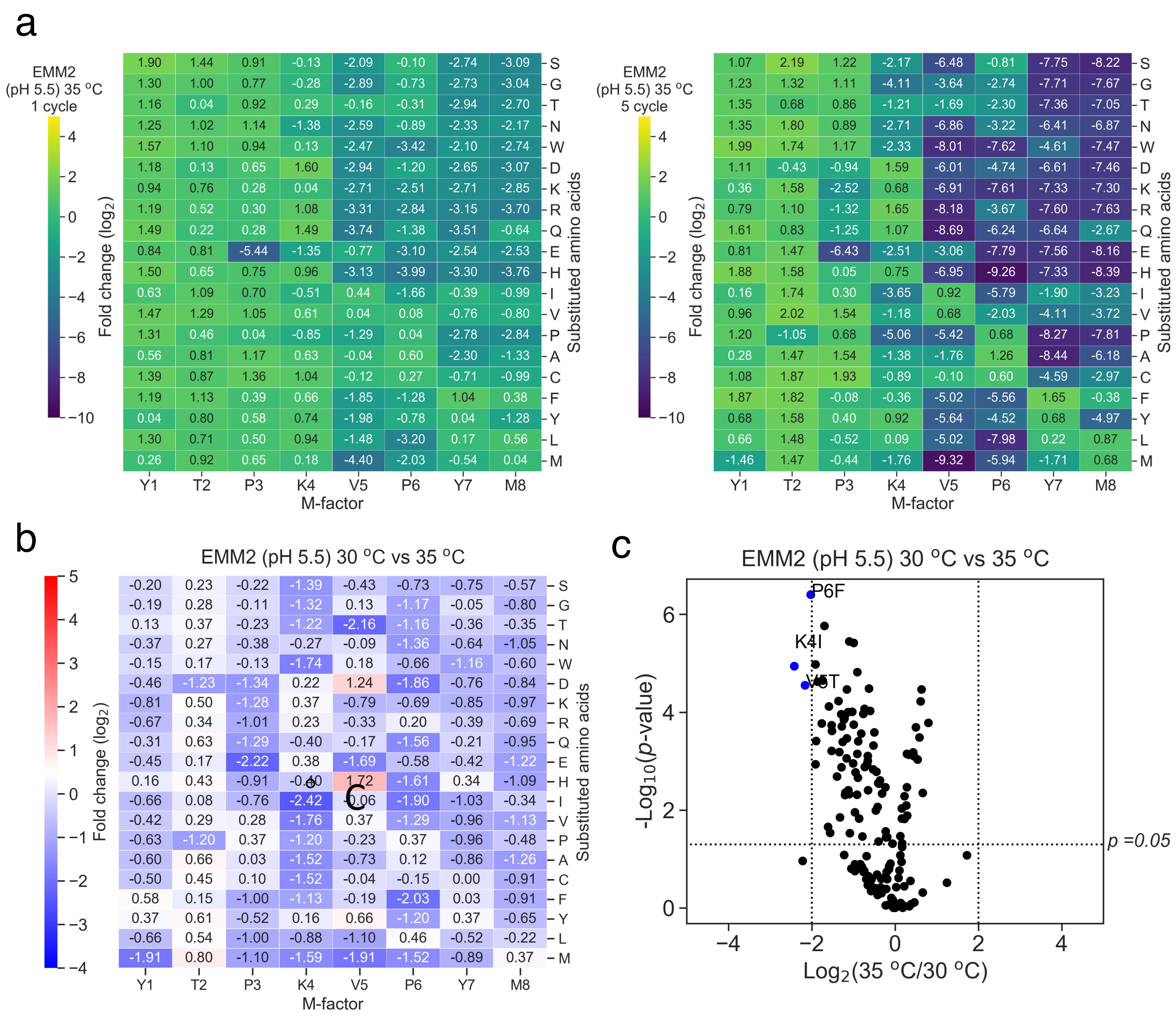

Supplementary Fig. 3 | Temperature-dependent selection dynamics of M-factor variants.

(a) Heatmaps showing $\log_2$ fold change in strain abundance after cycle 1 and 5 on EMM2 (pH 5.5) at 35 °C. (b) Heatmaps showing $\log_2$ fold change after 5 cycles comparing 30 °C vs 35 °C. (c) Volcano plot for the comparison in (b), highlighting variants with $|\log_2 \mathrm{FC}| \geq 2$ and $p < 0.05$.

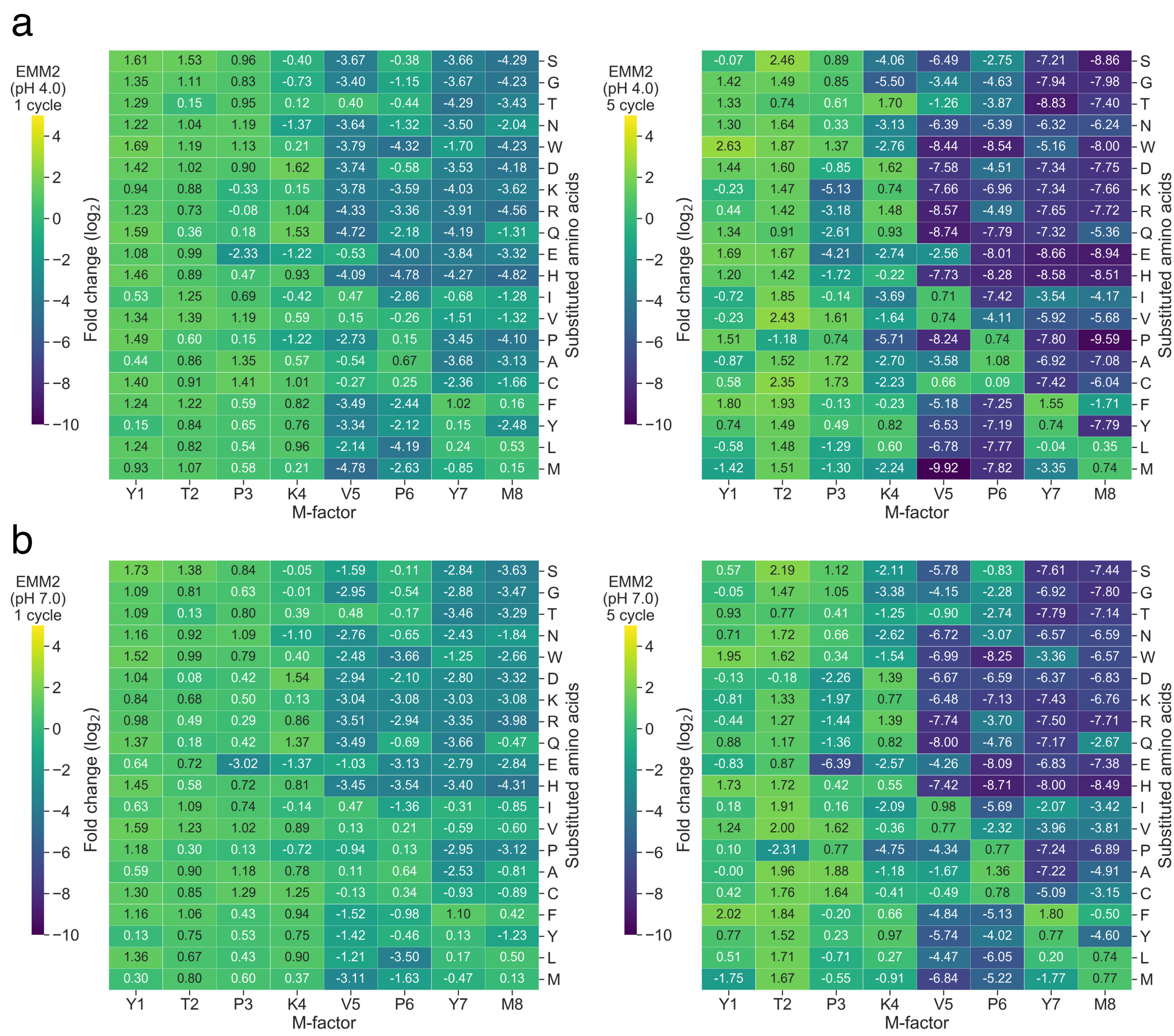

Supplementary Fig. 4 | Competition outcomes on EMM2 (pH 4.0) and EMM (pH 7.0).

Heatmaps showing $\log_2$ fold change in strain abundance after mating–sporulation cycles 1 and 5 on (a) EMM2 (pH 4.0) and (b) EMM2 (pH 7.0).

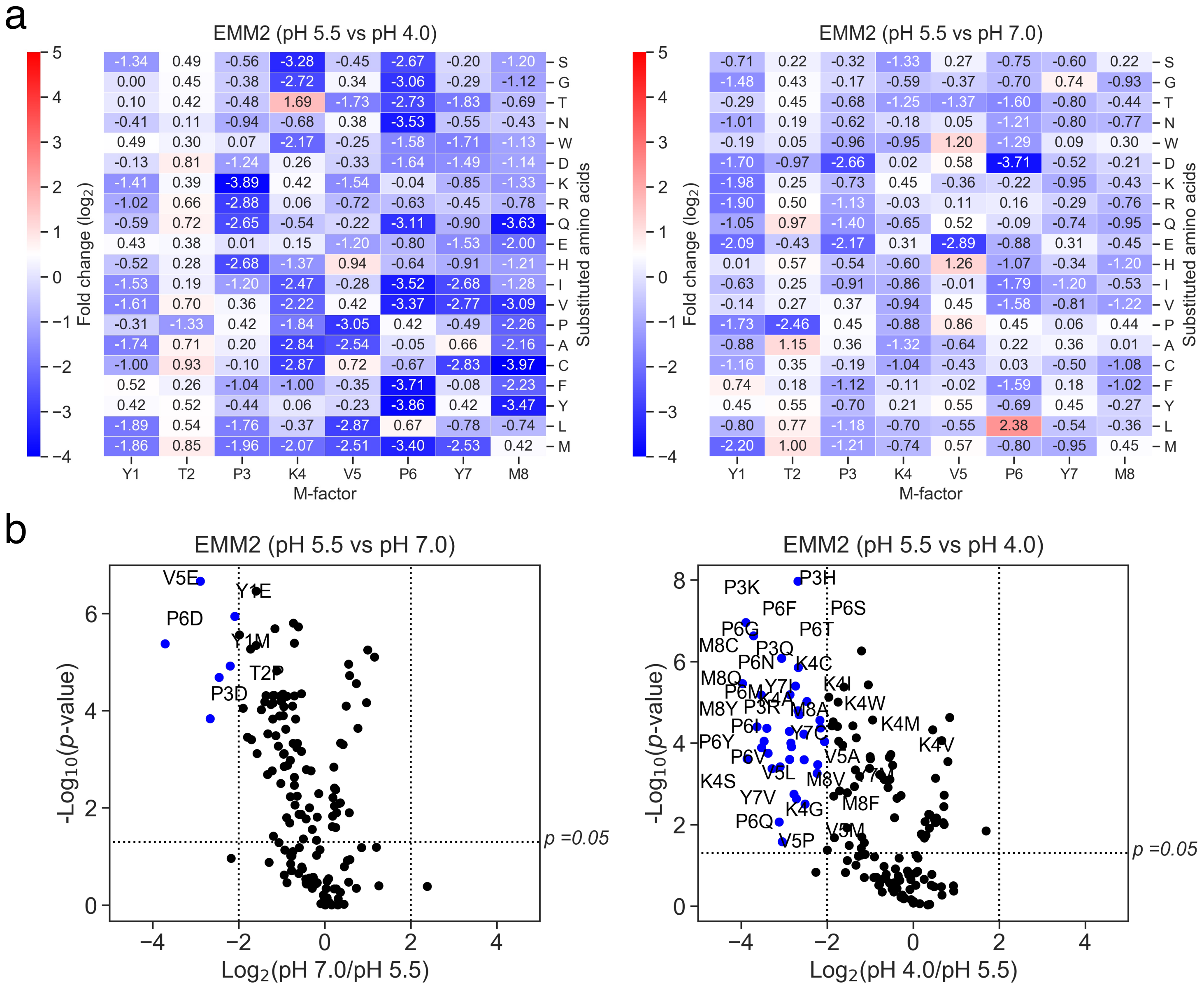

Supplementary Fig. 5 | pH-dependent selection dynamics of M-factor variants.

(a) Heatmaps showing $\log_2$ fold change after 5 cycles for EMM2 (pH 5.5 vs pH 4.0) and EMM2 (pH 5.5 vs pH 7.0). (b) Volcano plots for comparisons in (a), highlighting variants with $|\log_2 \text{FC}| \geq 2$ and $p < 0.05$.

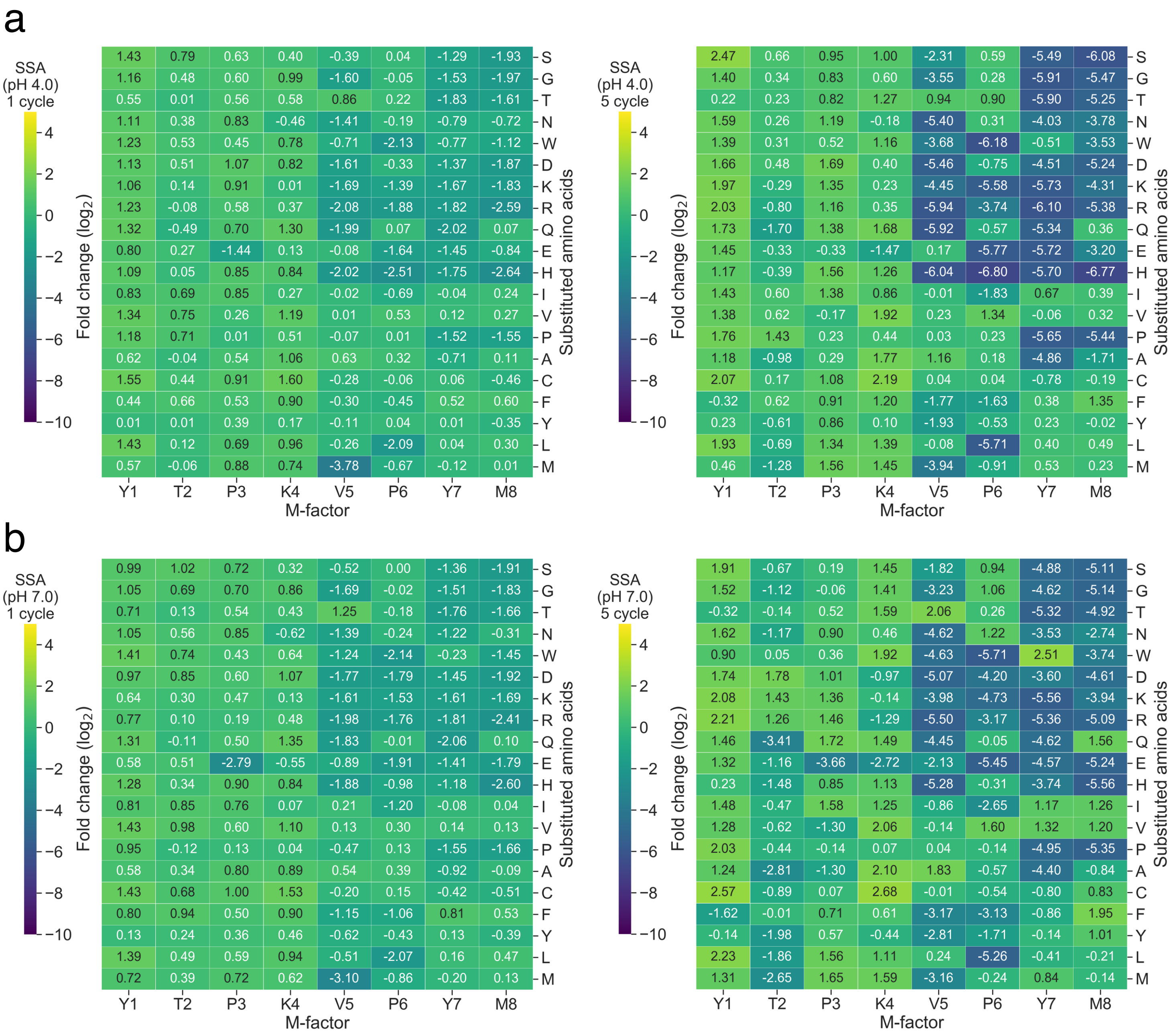

Supplementary Fig. 6 | Competition outcomes on SSA (pH 4.0) and SSA (pH 7.0).

Heatmaps showing log_2 fold change in strain abundance after mating–sporulation cycles 1 and 5 on (a) SSA (pH 4.0) and (b) SSA (pH 7.0).

Supplementary Fig. 7

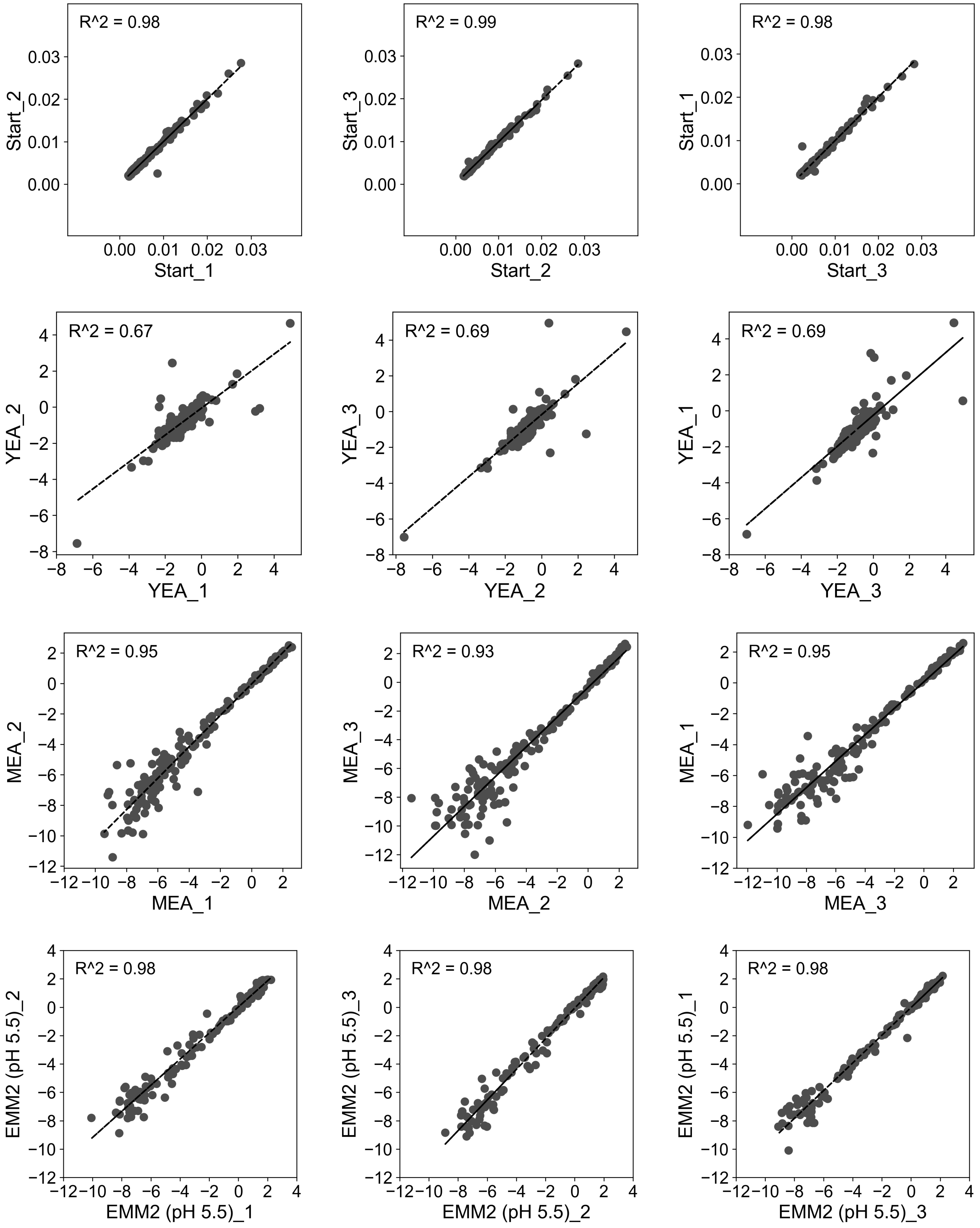

Supplementary Fig. 7 (continued)

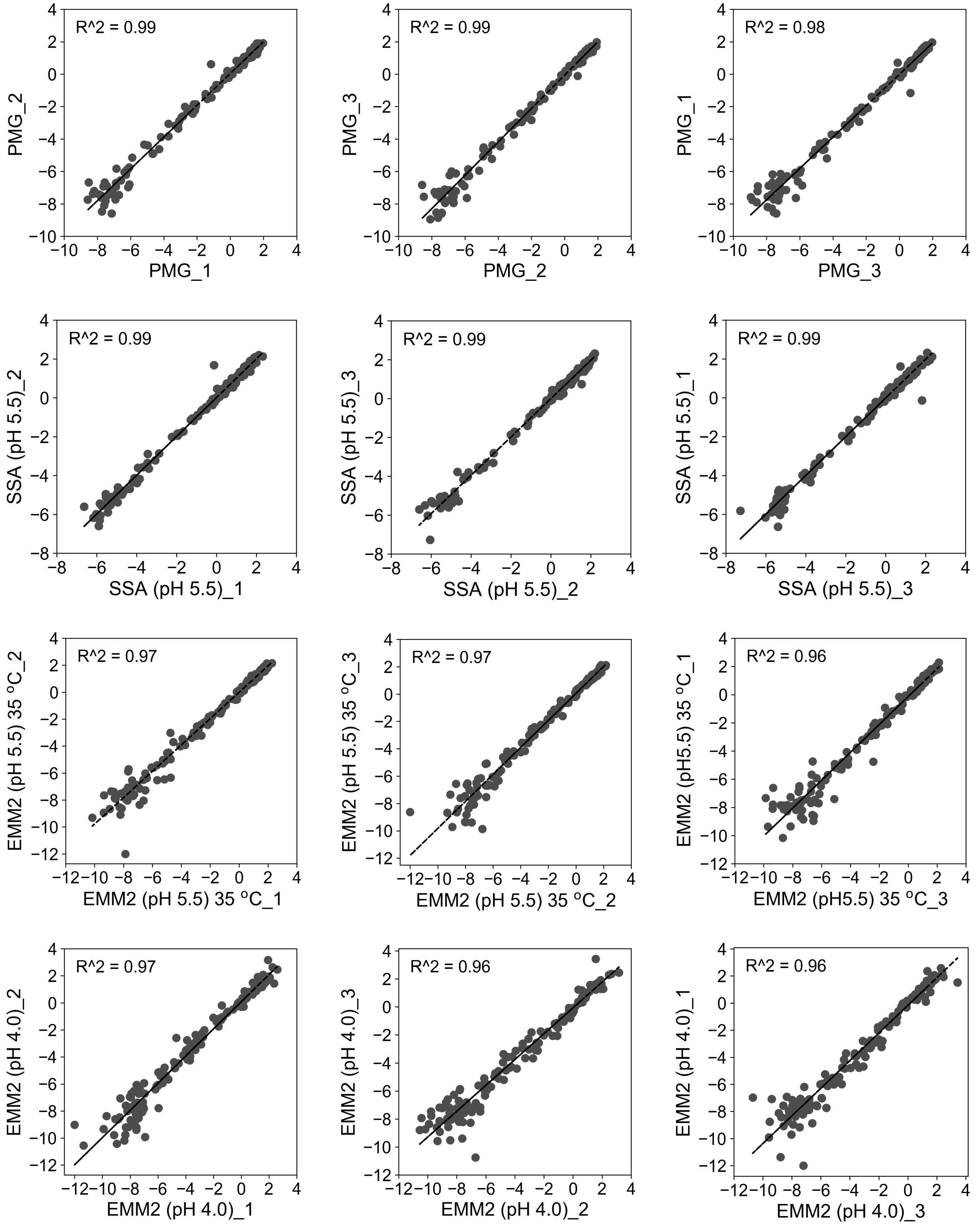

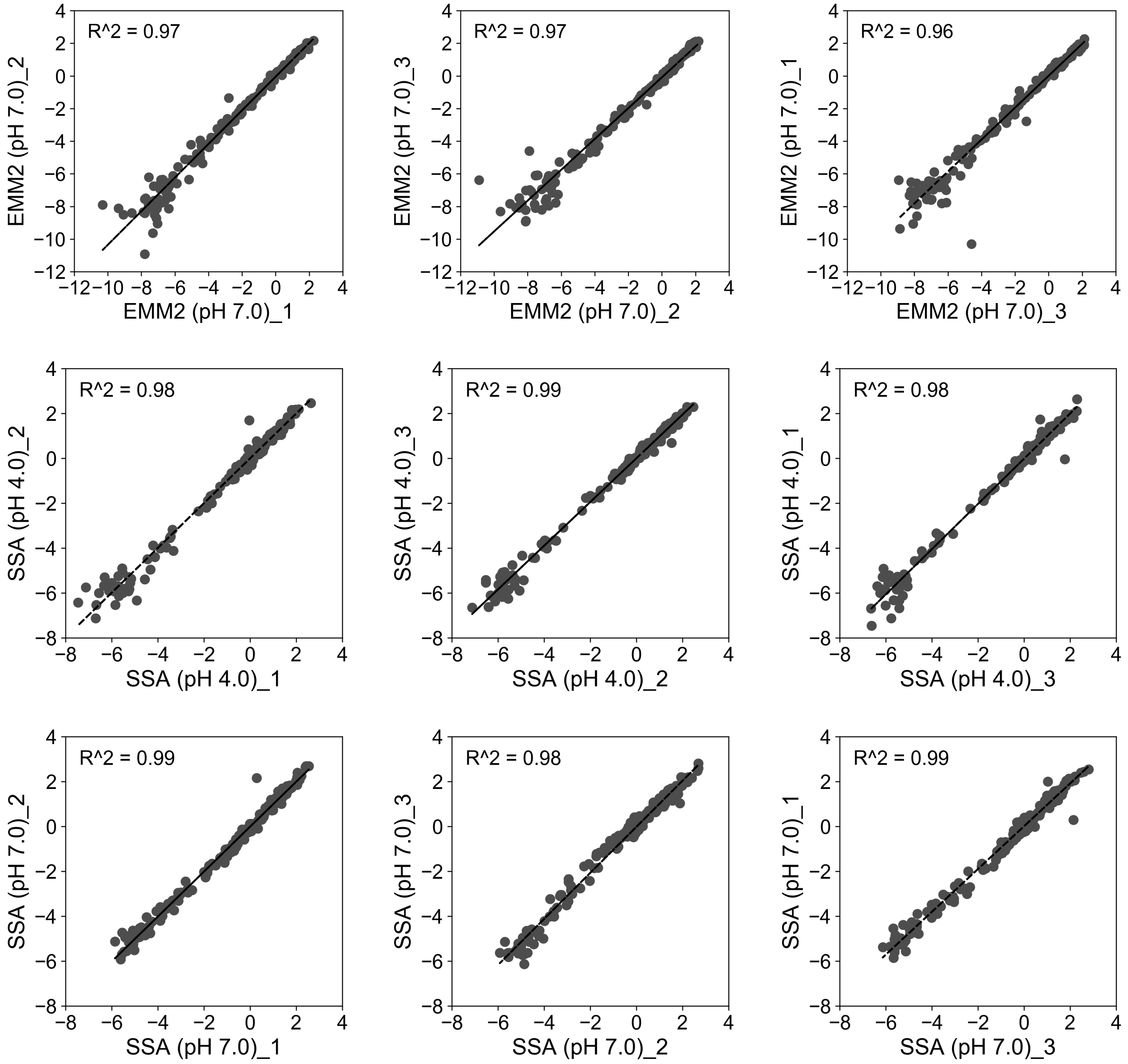

Supplementary Fig. 7 | Reproducibility of the high-throughput competition assay.

Pairwise scatter plots comparing $\log_2$ fold changes of all 153 strains between independent replicates. Each dot represents one strain; the diagonal line indicates y = x. High R^2 values demonstrate good reproducibility.

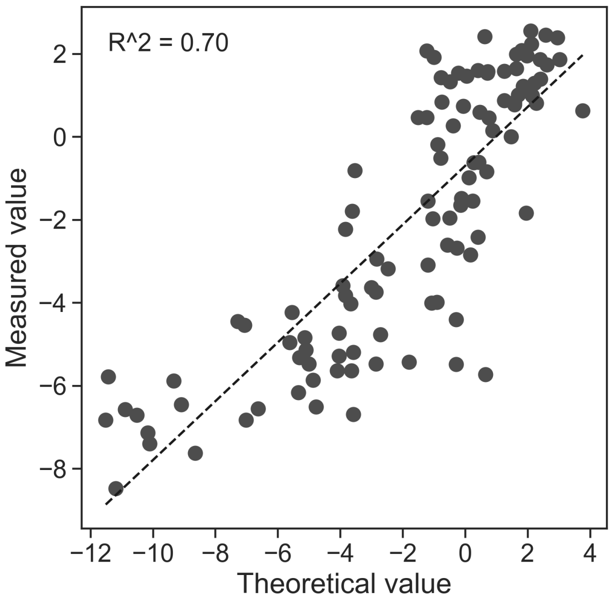

Supplementary Fig. 8 | Correlation between observed and predicted strain abundances.

Scatter plot comparing observed abundances after five mating cycles with predicted values based on previously measured single-strain mating frequencies[11]. A positive correlation ($R^2 = 0.70$) indicates minimal impact on relative fitness in the mixed population assay.

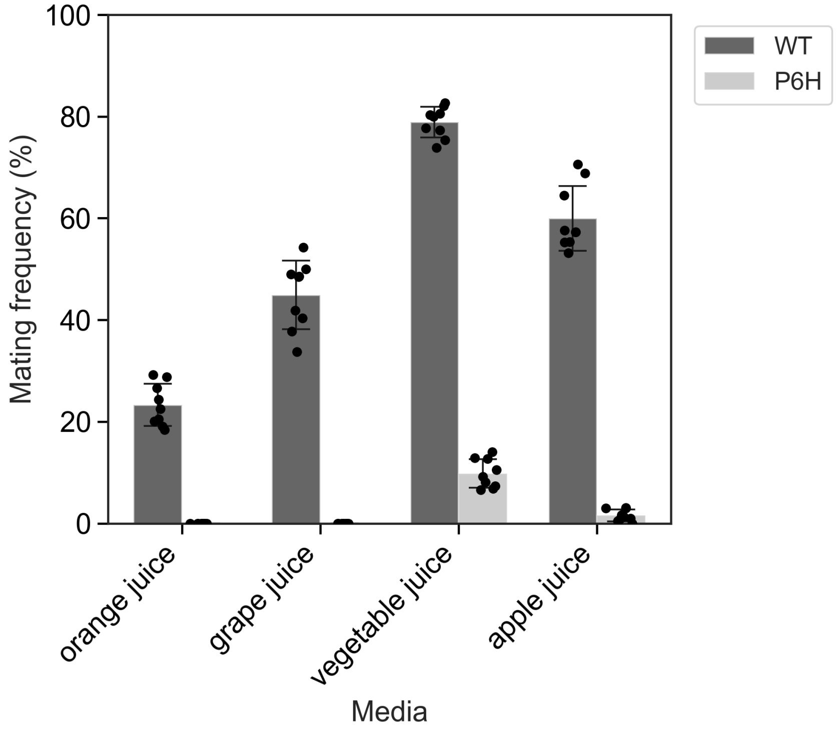

Supplementary Fig. 9 | Mating efficiency of P6H on fruit- and vegetable-derived media.

Mating frequencies of WT and P6H on grape, orange, apple, and vegetable juices. P6H shows sporulation on apple and vegetable juices, consistent with its activation at higher pH.

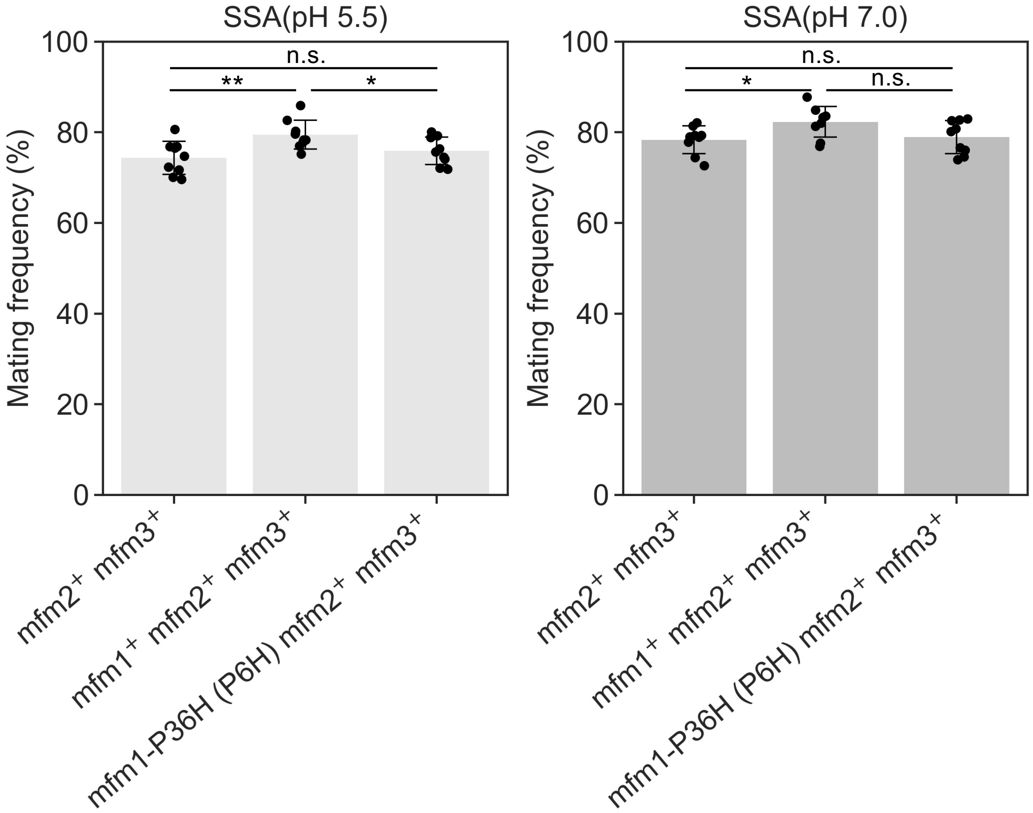

Supplementary Fig. 10 | Effect of P6H in a wild-type M-factor background.

The mating efficiencies of strains harboring two wild-type M-factor genes (*mfm2*$^+$ and *mfm3*$^+$; TS307) or carrying an additional wild-type *mfm1*$^+$ (TS1373) or *mfm1-P36H* (TS1374) gene at the *ade6* locus of TS307 were measured on SSA at pH 5.5 and pH 7.0. No significant differences were observed among the strains under either condition, indicating that the P6H variant did not interfere with mating driven by the endogenous wild-type pheromone genes. Welch's *t*-test: $*p < 0.05$; $**p < 0.01$; n.s., not significant.

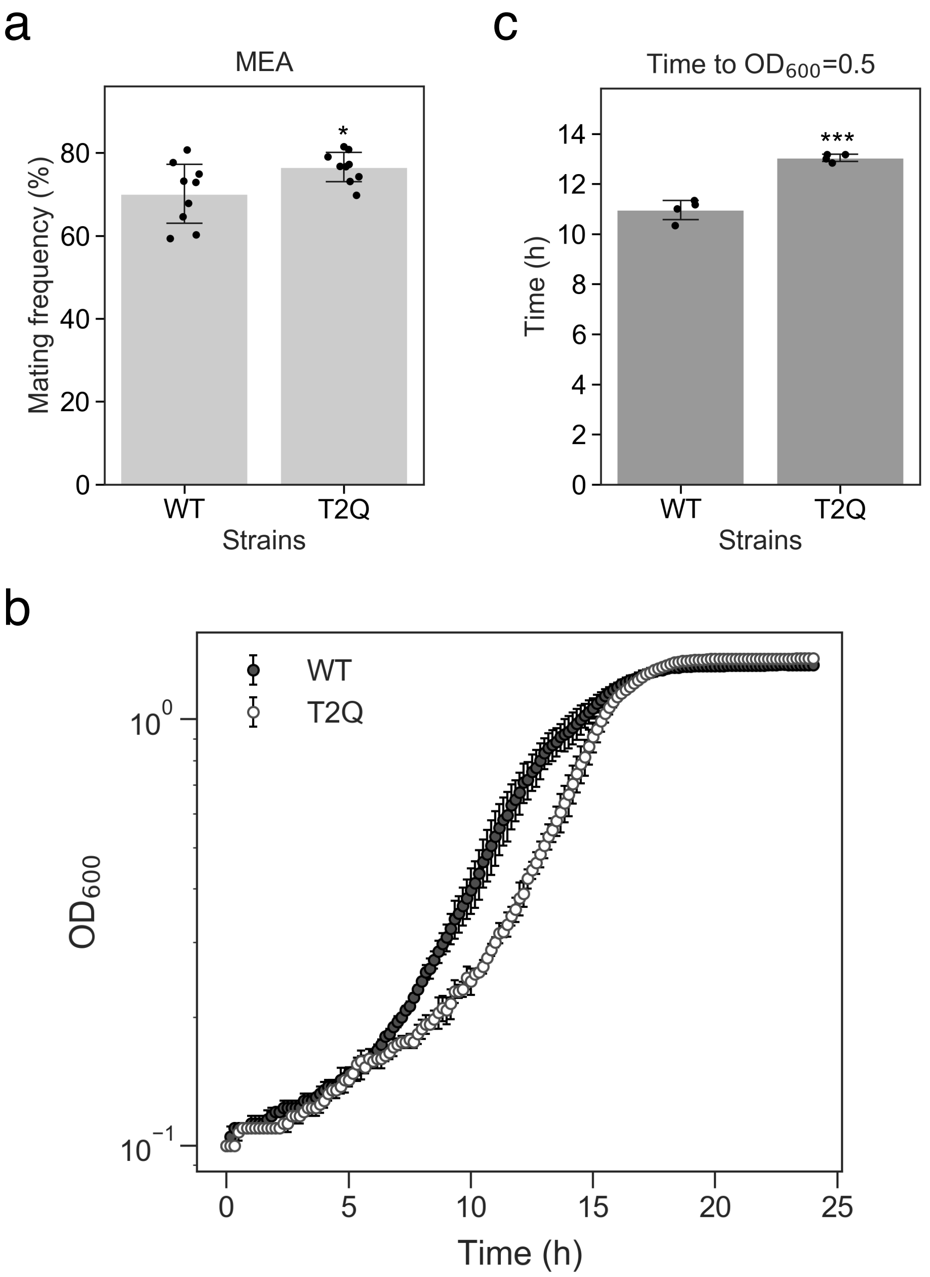

Supplementary Fig. 11 | Single-strain quantification of mating efficiency and vegetative growth reveals an intrinsic fitness trade-off in the T2Q variant.

(a) Mating efficiency of WT and the T2Q strains measured under single-strain conditions on MEA. The T2Q variant exhibits higher mating efficiency than WT, consistent with its enrichment under mating-permissive conditions in pooled competition assays. (b) Growth curves of WT and T2Q strains during vegetative growth in rich medium (YEL). OD_{600} was measured every 10 min for 24 h (n = 4 biological replicates), and mean ± s.d. is shown. The y-axis is plotted on a logarithmic scale. (c) Time required to reach OD_{600} = 0.5 during vegetative growth in YEL. The T2Q variant shows a significantly prolonged lag before reaching this threshold compared with WT. Welch's t-test: *p <0.05; ***p <0.001.
