# Supplementary material for: Context-dependent activation and evolutionary buffering of a mating pheromone in fission yeast
Source: Commun Biol. 2026 Apr 21;9:534. doi: 10.1038/s42003-026-10058-6 (PMC13099959; doi:10.1038/s42003-026-10058-6)
Supplement: Supplementary file 9 — Description of Additional Supplementary File [file 42003_2026_10058_MOESM9_ESM.docx]

**Description of Additional Supplementary File**File name: Supplementary data 1
Description: Strains used and constructed in this study.

File name: Supplementary data 2
Description: Primers used for plasmid construction and sequencing.

File name: Supplementary data 3
Description: Normalized read count data for all 153 variants across multiple media, pH, and temperature conditions.

File name: Supplementary data 4
Description: Microscopy-based quantification of mating efficiency, including all replicate counts and field-level scores.

File name: Supplementary data 5
Description: The optical density at 600 nm (OD600) was measured every 10 min for the WT and T2Q strains during 24 h of growth in YEL medium using a 96-well microplate.

File name: Supplementary data 6
Description: Plasmids used and constructed in this study.

File name: Supplementary data 7
Description: Source data for Fig. 3a, 3c, 3d, 4a, 4b, 5a, 5b.
