# Supplementary material for: Context-dependent activation and evolutionary buffering of a mating pheromone in fission yeast
Source: Commun Biol. 2026 Apr 21;9:534. doi: 10.1038/s42003-026-10058-6 (PMC13099959; doi:10.1038/s42003-026-10058-6)
Supplement: Supplementary file 10 — Reporting Summary [file 42003_2026_10058_MOESM10_ESM.pdf]

nature portfolio

Corresponding author(s): Taisuke Seike

Last updated by author(s): Sep 26, 2025

Reporting Summary

Nature Portfolio wishes to improve the reproducibility of the work that we publish. This form provides structure for consistency and transparency in reporting. For further information on Nature Portfolio policies, see our Editorial Policies and the Editorial Policy Checklist.

Statistics

For all statistical analyses, confirm that the following items are present in the figure legend, table legend, main text, or Methods section.

n/a	Confirmed	
☐	☒	The exact sample size (*n*) for each experimental group/condition, given as a discrete number and unit of measurement
☐	☒	A statement on whether measurements were taken from distinct samples or whether the same sample was measured repeatedly
☐	☒	The statistical test(s) used AND whether they are one- or two-sided<br>*Only common tests should be described solely by name; describe more complex techniques in the Methods section.*
☐	☒	A description of all covariates tested
☐	☒	A description of any assumptions or corrections, such as tests of normality and adjustment for multiple comparisons
☐	☒	A full description of the statistical parameters including central tendency (e.g. means) or other basic estimates (e.g. regression coefficient) AND variation (e.g. standard deviation) or associated estimates of uncertainty (e.g. confidence intervals)
☐	☒	For null hypothesis testing, the test statistic (e.g. F, t, r) with confidence intervals, effect sizes, degrees of freedom and P value noted<br>*Give P values as exact values whenever suitable.*
☒	☐	For Bayesian analysis, information on the choice of priors and Markov chain Monte Carlo settings
☐	☒	For hierarchical and complex designs, identification of the appropriate level for tests and full reporting of outcomes
☐	☒	Estimates of effect sizes (e.g. Cohen's d, Pearson's r), indicating how they were calculated

Our web collection on statistics for biologists contains articles on many of the points above.

Software and code

Policy information about availability of computer code

Data collection	No software was used by the authors for data collection. Illumina MiSeq sequencing was performed by the core facility, which provided demultiplexed FASTQ files already separated by sample-specific barcodes. Other experimental data (microscopy-based mating efficiency counts, β-galactosidase assays) were recorded manually.
Data analysis	Open-source software was used for all analyses:<br>seqkit (v2.10.1) for sequence processing,<br>pandas (v2.2.3) and numpy (v2.2.5) for data handling and numerical analyses,<br>matplotlib (v3.10.3) and seaborn (v0.13.2) for visualization,<br>scipy (v1.15.3) for statistical analyses.<br>All custom scripts are available upon reasonable request from the corresponding author.

For manuscripts utilizing custom algorithms or software that are central to the research but not yet described in published literature, software must be made available to editors and reviewers. We strongly encourage code deposition in a community repository (e.g. GitHub). See the Nature Portfolio guidelines for submitting code & software for further information.

Data

Policy information about availability of data

All manuscripts must include a data availability statement. This statement should provide the following information, where applicable:

- Accession codes, unique identifiers, or web links for publicly available datasets
- A description of any restrictions on data availability
- For clinical datasets or third party data, please ensure that the statement adheres to our policy

All data supporting the findings of this study are available within the article, figures, and Supplementary Data files. Raw sequencing counts, processed datasets, and numerical values underlying graphs are provided in Supplementary Data. No restrictions apply to data availability.

Research involving human participants, their data, or biological material

Policy information about studies with human participants or human data. See also policy information about sex, gender (identity/presentation), and sexual orientation and race, ethnicity and racism.

Reporting on sex and gender	Not applicable
Reporting on race, ethnicity, or other socially relevant groupings	Not applicable
Population characteristics	Not applicable
Recruitment	Not applicable
Ethics oversight	Not applicable

Note that full information on the approval of the study protocol must also be provided in the manuscript.

Field-specific reporting

Please select the one below that is the best fit for your research. If you are not sure, read the appropriate sections before making your selection.

☐ Life sciences ☐ Behavioural & social sciences ☒ Ecological, evolutionary & environmental sciences

For a reference copy of the document with all sections, see nature.com/documents/nr-reporting-summary-flat.pdf

Ecological, evolutionary & environmental sciences study design

All studies must disclose on these points even when the disclosure is negative.

Study description	This study investigates how sequence variation in the fission yeast Schizosaccharomyces pombe mating pheromone (M-factor) influences reproductive success under different environmental conditions. Using a comprehensive single–amino acid substitution library, we combined pooled competition assays, microscopy-based mating efficiency assays, and synthetic peptide reporter assays to test how environmental pH and mutations shape pheromone activity and evolutionary robustness.
Research sample	The research sample consisted of S. pombe h90 strains carrying all 152 single–amino acid variants of the nine–amino acid M-factor peptide, plus the wild type strain (total = 153 genotypes). Additional assays included synthetic peptides and comparative constructs incorporating substitutions from the related species S. octosporus.
Sampling strategy	All variants were included without prior filtering; no sample-size calculation was performed, as the design was comprehensive rather than selective. Each genotype was assayed in pooled competition (n = 3 biological replicates), as well as in targeted single-strain validation assays.
Data collection	Variant abundance was quantified by amplicon sequencing of pooled competition samples at cycles 1 and 5. Mating efficiencies were assessed microscopically by scoring >1,000 cells per condition. Receptor activation was assayed with a map4–lacZ β-galactosidase reporter using synthetic peptides.
Timing and spatial scale	Competition assays were performed over five iterative mating–sporulation–germination cycles (~6 days per experiment). pH and temperature manipulations were performed under controlled laboratory conditions. Juice-based media were derived from commercially available grape, orange, apple, and vegetable juices. No field sampling was conducted.
Data exclusions	No data were excluded. All replicates and genotypes were retained in the analysis.
Reproducibility	All experiments were performed in three independent biological replicates, which showed high reproducibility ($R^2 > 0.9$ in replicate

Reproducibility	comparisons). Representative microscopy and reporter assays were repeated at least three times with consistent results.
Randomization	For pooled competition assays, all 153 genotypes were combined at equal initial abundance (~1×10^5 cells per strain). For microscopy-based assays, fields of view were selected randomly without bias.
Blinding	Blinding was not relevant because outcomes (variant abundance, sporulation efficiency, reporter activity) were objectively measured via sequencing, microscopy counts, or enzymatic activity. However, microscopy counts were performed without reference to strain identity.

Did the study involve field work? ☐ Yes ☒ No

Reporting for specific materials, systems and methods

We require information from authors about some types of materials, experimental systems and methods used in many studies. Here, indicate whether each material, system or method listed is relevant to your study. If you are not sure if a list item applies to your research, read the appropriate section before selecting a response.

Materials & experimental systems

n/a	Involved in the study	
☒	☐	Antibodies
☒	☐	Eukaryotic cell lines
☒	☐	Palaeontology and archaeology
☐	☒	Animals and other organisms
☒	☐	Clinical data
☒	☐	Dual use research of concern
☒	☐	Plants

Methods

n/a	Involved in the study	
☒	☐	ChIP-seq
☒	☐	Flow cytometry
☒	☐	MRI-based neuroimaging

Animals and other research organisms

Policy information about studies involving animals; ARRIVE guidelines recommended for reporting animal research, and Sex and Gender in Research

Laboratory animals	No laboratory animals were used.
Wild animals	No wild animals were used.
Reporting on sex	Not applicable. The study used unicellular fungi (Schizosaccharomyces pombe and S. octosporus), which do not have separate sexes but distinct mating types (Plus and Minus).
Field-collected samples	Not applicable. All yeast strains were laboratory strains; no field-collected samples were performed in this study.
Ethics oversight	Not applicable. The research involved non-pathogenic yeast and did not require institutional ethics approval.

Note that full information on the approval of the study protocol must also be provided in the manuscript.

Plants

Seed stocks	No seed stocks were used.
Novel plant genotypes	No plants were used.
Authentication	Not applicable.
